# Azobenzene DNA Intercalator/Cyclodextrin Pseudo‐Rotaxane: From Photoswitchable Chirality and Fluorescence to DNA Melting Control

**DOI:** 10.1002/open.202500382

**Published:** 2025-08-20

**Authors:** Olivier Abodja, Astrid Walrant, Sergii Rudiuk, Mathieu Morel, Damien Baigl

**Affiliations:** ^1^ CPCV Department of Chemistry Ecole Normale Supérieure PSL University, Sorbonne Université, CNRS 75005 Paris France

**Keywords:** DNA hybridization, DNA nanotechnology, host‐guest, photoswitch, supramolecular chemistry

## Abstract

Pseudo‐rotaxanes are reversibly interlocked molecules with at least one linear molecule threaded into a macrocycle and, contrary to rotaxanes, an advantageous ability to be dissociated. Cyclodextrins constitute attracting macrocyclic host entities to build such dynamic structures for their oligosaccharide nature, conic shape, amphiphilic character and biocompatibility. Here we show that using an azobenzene DNA intercalator as a guest allows to build a pseudo‐rotaxane combining several remarkable properties, including light‐controlled assembly/disassembly, photoreversible chirality and fluorescence, as well as the capability to affect the melting temperature of double‐stranded DNA through intercalator host–guest complexation.

## Graphical abstract

We show that using an azobenzene DNA intercalator (AzoDiGua) as a guest for a cyclodextrin (α‐CD) host cavity results in the formation of a pseudo‐rotaxane combining several remarkable properties, such as light‐controlled assembly/disassembly, photoreversible chirality and fluorescence, as well as the capability to control the melting temperature of double‐stranded DNA through intercalator host–guest complexation.

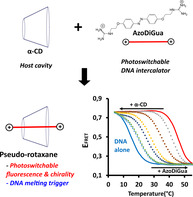

## 1 Introduction

The design and construction of molecular systems capable of motion and complex reconfiguration at the molecular scale under the actuation of an external stimulus have attracted considerable attention, especially in the context of building functional molecular machines.^[^ ^1^ ^]^ For this purpose, interlocked architectures have been widely explored due to the precise control of their geometry.^[^ ^1^, ^2^, ^3^, ^4^ ^]^ Among them, host–guest systems built up between azobenzene derivatives and cyclodextrins have been investigated. Indeed, on the one hand, cyclodextrins are interesting host oligosaccharide molecules due to their cone‐like structure, their amphiphilic properties and their biocompatibility.^[^ ^5^, ^6^, ^7^ ^]^ On the other hand, azobenzene derivatives are stable and robust components able to reversibly photoisomerize.^[^ ^8^ ^]^ The combination of these two bricks enabled the construction of a variety of smart photoswitchable functional host–guest systems.^[^ ^3^, ^9^, ^10^, ^11^, ^12^, ^13^ ^]^ Pseudo‐rotaxanes are particularly interesting for their ability to be dissociated, contrary to interlocked rotaxanes bearing bulky stoppers.^[^ ^14^ ^]^ Using light to control such association/dissociation mechanism thus appears as a valuable objective but only few functional azobenzene‐cyclodextrin pseudo‐rotaxanes have been reported so far.^[^ ^1^, ^2^, ^3^, ^4^, ^5^, ^6^ ^]^

Using host–guest systems to control the properties of a target is of particular interest, as it does not imply to chemically alter the target. In recent years, DNA has turned out to be an interesting target as it constitutes a highly programmable building block for bottom‐up nanotechnology through Watson–Crick base pairing.^[^ ^15^, ^16^, ^17^, ^18^, ^19^, ^20^ ^]^ Hence, controlling DNA hybridization is of great interest to trigger a broad range of biological or supramolecular processes based on DNA assembly and disassembly.^[^ ^21^, ^22^ ^]^ Usually, temperature is used as an external stimulus to trigger DNA melting preventing applications in isothermal conditions. Looking for alternative stimuli is thus particularly relevant in order to control DNA hybridization at constant temperature. For instance, light has been widely investigated as a physical stimulus to isothermally photocontrol DNA melting through photoswitchable molecules incorporated in double stranded DNA.^[^ ^23^, ^24^, ^25^, ^26^, ^27^ ^]^ This can be conveniently achieved using a photosensitive intercalator, such as AzoDiGua, an azobenzene‐based bolaform amphiphilic photochrome bearing two guanidinium moieties, which was demonstrated to act as a photoregulator of DNA melting.^[^ ^23^ ^]^ In this work, we propose a new strategy to trigger DNA melting at constant temperature, based not on the photochemical properties of AzoDiGua but on its geometric and amphiphilic characteristics. For this purpose, we investigated the possibility to use the competitive AzoDiGua insertion in the hydrophobic cavity of cyclodextrins as a supramolecular stimulus to control DNA intercalation and thus its hybridization at constant temperature.

To our knowledge, while cyclodextrin‐responsive DNA compaction has already been reported,^[^ ^28^ ^]^ cyclodextrin‐responsive DNA intercalation has never been studied so far. In this work, we show that AzoDiGua is able to form a robust pseudo‐rotaxane with an α‐cyclodextrin. This host–guest system was found to be photoswitchable, resulting in precise photocontrol over its physical properties, including complexation‐induced fluorescence and chirality. This new kind of pseudo‐rotaxane was used as a supramolecular trigger to dynamically regulate DNA intercalation and tune DNA melting temperature.

## 2 Results and Discussion

### 2.1 Building a Photoswitchable Pseudo‐Rotaxane with Multiple Properties

**Figure 1:**
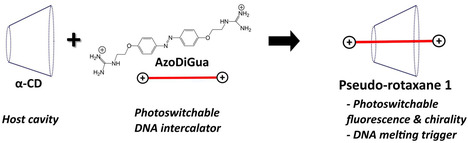
Formation of a multifunctional pseudo‐rotaxane by host–guest complexation between a cyclodextrin and an azobenzene DNA intercalator. Alpha‐cyclodextrin (α‐CD) is used as a cavity to host AzoDiGua, a photoswitchable DNA intercalator, forming the pseudo‐rotaxane **1** with photoswitchable fluorescence and chirality. This complexation can be exploited as a supramolecular trigger to control DNA melting properties.

Our concept is depicted in **Figure** **1**. It consists in co‐assembling alpha‐cyclodextrin (α‐CD), acting as a host cavity, with AzoDiGua, an azobenzene dicationic DNA intercalator, to form the pseudo‐rotaxane **1**. We hypothesized that this complexation would result in the confinement‐induced fluorescence of the complexed azobenzene moiety as well as a chirality transfer upon incorporation of AzoDiGua in the α‐CD cavity. Having a photoswitchable AzoDiGua/α‐CD complexation would thus result in a photocontrol of both properties. Finally, AzoDiGua intercalation into DNA being accompanied by a steep increase of the DNA melting temperature (*T*_m_),^[^ ^23^ ^]^ the capacity of α‐CD to complex AzoDiGua could be used as a supramolecular *T*_m_ regulator and a trigger to control DNA melting properties.

### 2.2 AzoDiGua/α‐CD Complexation and Structural Properties

**Figure 2:**
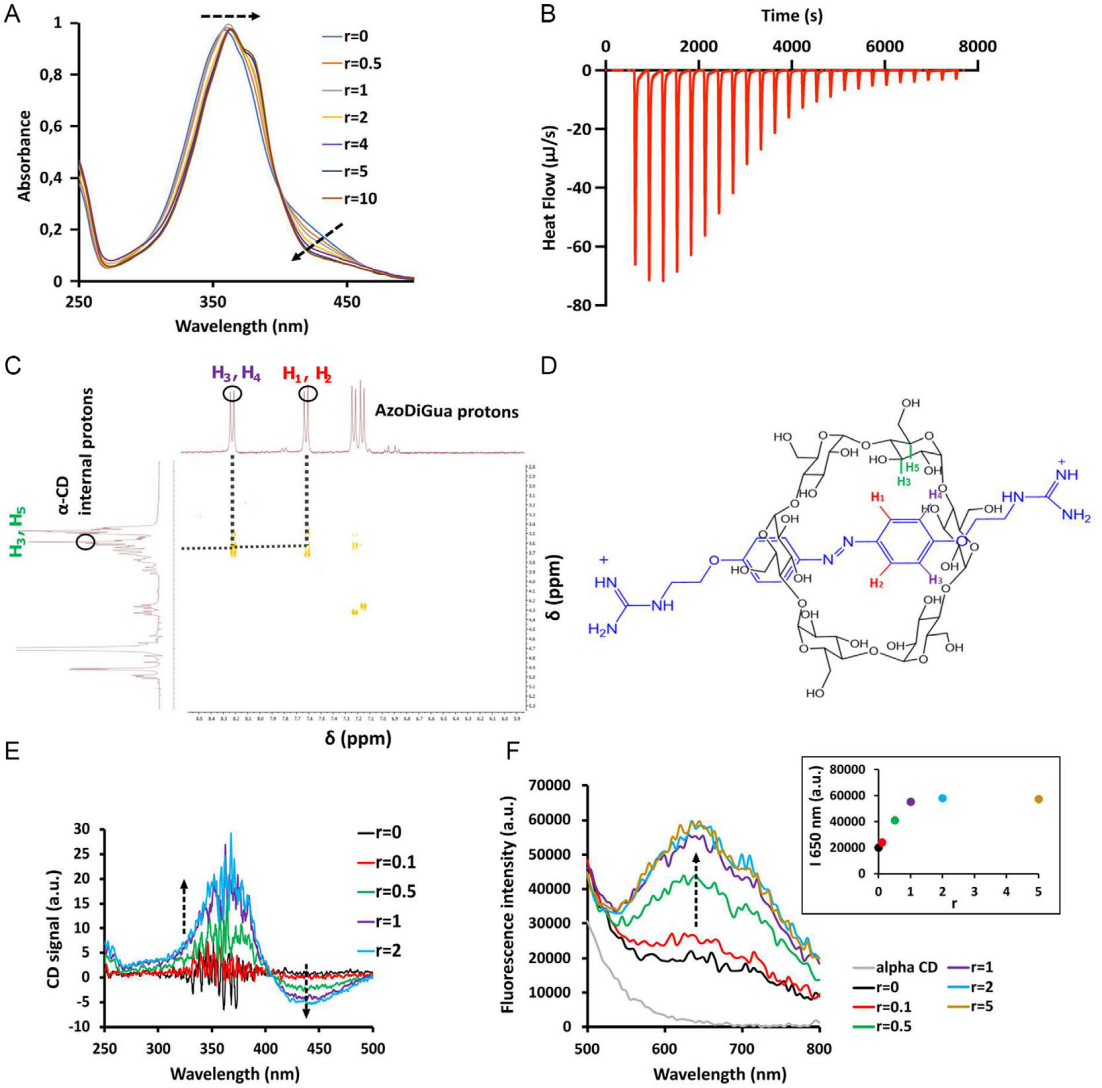
Structure, chirality transfer and fluorescence of pseudo‐rotaxane **1**. A) UV–vis absorbance spectra of AzoDiGua (50 μM) with increasing ratios *r* of α‐CD (*r* = [α‐CD]/[AzoDiGua]). B) Isothermal titration calorimetry (ITC) thermogram of the titration of AzoDiGua (1 mL of 1 mM solution) by α‐CD (10 μL injections of 10 mM solutions) at *T* = 25 °C. C) 2D ROESY ^1^H NMR spectrum (D_2_O, 300 MHz) of an equimolar mixture of AzoDiGua and α‐CD ([AzoDiGua] = [α‐CD] = 5 mM), with peak assignments for H1–H4 AzoDiGua protons and H3,H5 α‐CD protons. D) Proposed structure of the complex with the assigned protons shown in (C). E) Circular dichroism titration of AzoDiGua (500 μM) with increasing ratios *r* of α‐CD. F) *Main graph:* fluorescence emission spectra (excitation 400 nm) of α‐CD alone (500 μM, grey curve) and AzoDiGua (500 μM) with increasing ratios *r* of α‐CD. *Inset:* fluorescence emission intensity at 650 nm (excitation 400 nm) of AzoDiGua (500 μM) as a function of *r*. In all graphics, dashed arrows indicate increasing values of *r*. All experiments were done in Tris HCl buffer (50 mM) except for NMR analyses (C–D) where D_2_O was used.

In order to probe the interaction between AzoDiGua and α‐CD, UV–visible titration of AzoDiGua by α‐CD was performed (**Figure** **2**A). The *trans‐*AzoDiGua π–π* absorption band gradually moved from 359 to 365 nm upon progressive addition of α‐CD. This bathochromic effect is attributed to the decrease of the polarity of the micro‐environment around the azobenzene groups^[^ ^29^ ^]^ upon the progressive incorporation of *trans*‐AzoDiGua molecules in the hydrophobic cavity of α‐CD resulting in the formation of pseudo‐rotaxane **1**. Isothermal titration calorimetry (ITC) evidenced a favorable exothermic interaction upon adding increasing amounts of α‐CD to AzoDiGua at 25 °C (Figure 2B), leading to the formation of complexes with a dissociation constant *K*_D_ = 5.7 × 10^−5^ M, a stoichiometry 1:1 and a binding enthalpy Δ*H* = −25 kJ mol^−1^ (Figure S1, Supporting Information). Replacing α‐CD with β‐CD resulted in a less thermodynamically favorable complexation with AzoDiGua (*K*_D_ = 1.4 × 10^−4^ M, Δ*H *= −8 kJ mol^−1^, Figure S2, Supporting Information), attributed to the larger cavity size of β‐CD. NMR characterization was performed to gain structural insights into the respective arrangement of AzoDiGua and α‐CD upon complexation. 2D DOSY ^1^H NMR showed that the aromatic protons of AzoDiGua (7.05 and 7.75 ppm) had a higher diffusion coefficient for AzoDiGua alone (*D*_AzoDiGua_ = 3 × 10^−6^ m^2^ s^−1^, Figure S3, Supporting Information) than in presence of α‐CD (*D*_AzoDiGua/α‐CD_ = 1.9 × 10^−6^ m^2^ s^−1^, Figure S4, Supporting Information) in agreement with the formation of a larger‐size, slower‐diffusing complex when AzoDiGua was mixed with α‐CD. Moreover, 2D ROESY ^1^H NMR showed internuclear correlations between the aromatic protons of AzoDiGua and the internal protons of α‐CD (Figure 2C), demonstrating that the complex formed by the inclusion of AzoDiGua in the α‐CD cavity. ^1^H 1D NMR (Figure S5–S7, Supporting Information) showed that the aromatic protons signals of AzoDiGua (7.73 and 7.05 ppm, Figure S5, Supporting Information) appeared at a higher chemical shift in the presence of α‐CD (8.23 and 7.63 ppm, Figure S7, Supporting Information), in agreement with a change of polarity of the surrounding medium of the aromatic protons upon insertion of AzoDiGua in the α‐CD cavity. Furthermore, two new peaks in the aromatic regions appeared (7.24 and 7.17 ppm) probably corresponding to the aromatic protons of the second aromatic cycle of AzoDiGua (Figure S7, Supporting Information). This suggests that the two aromatic cycles of AzoDiGua are not equivalent anymore when included in α‐CD leading to a loss of the symmetry of the molecule. All these results demonstrate the formation of an asymmetric inclusion complex resulting in pseudo‐rotaxane **1** with the suggested structure shown in Figure 2D.

### 2.3 Inclusion‐Induced Chirality and Fluorescence

Circular dichroism measurements were performed to probe whether a chirality transfer occurred upon inclusion of achiral AzoDiGua in the α‐CD cavity. When α‐CD was added to AzoDiGua, a positive Cotton effect appeared at 365 nm and increased with increasing concentrations of α‐CD (Figure 2E). At this wavelength, which corresponds to the π–π* electronic transition of AzoDiGua, neither α‐CD nor the buffer adsorbed (Figure S8, Supporting Information), showing that the induced circular dichroism was due to the gradual inclusion of AzoDiGua in the increasing amounts of available α‐CD. Such a chirality transfer is explained by the cavity of α‐CD creating a chiral environment to AzoDiGua.^[^ ^30^, ^31^ ^]^

When excited at 400 nm, α‐CD alone did not display any significant fluorescence while AzoDiGua was weakly fluorescent with an emission maximum at around 650 nm. Notably, this AzoDiGua fluorescence got progressively enhanced in the presence of increasing amounts of α‐CD (Figure 2F). It was shown previously that blocking AzoDiGua conformation through co‐crystallization^[^ ^32^ ^]^ or aggregation^[^ ^33^ ^]^ led to a similar fluorescence increase due to the restriction of the azobenzene rotation/inversion relaxation mechanisms after photoexcitation.^[^ ^34^ ^]^ By analogy, we can attribute the fluorescence enhancement observed here through AzoDiGua/α‐CD complexation to the AzoDiGua inclusion in the cavity restricting the azobenzene conformation relaxation after photoexcitation. All these results not only confirmed the inclusion of AzoDiGua in α‐CD cavity but also demonstrated how this interaction resulted in chiral and fluorescent properties acquired by the resulting complex.

### 2.4 A Pseudo‐Rotaxane with Photoswichable Chirality and Fluorescence

**Figure 3:**
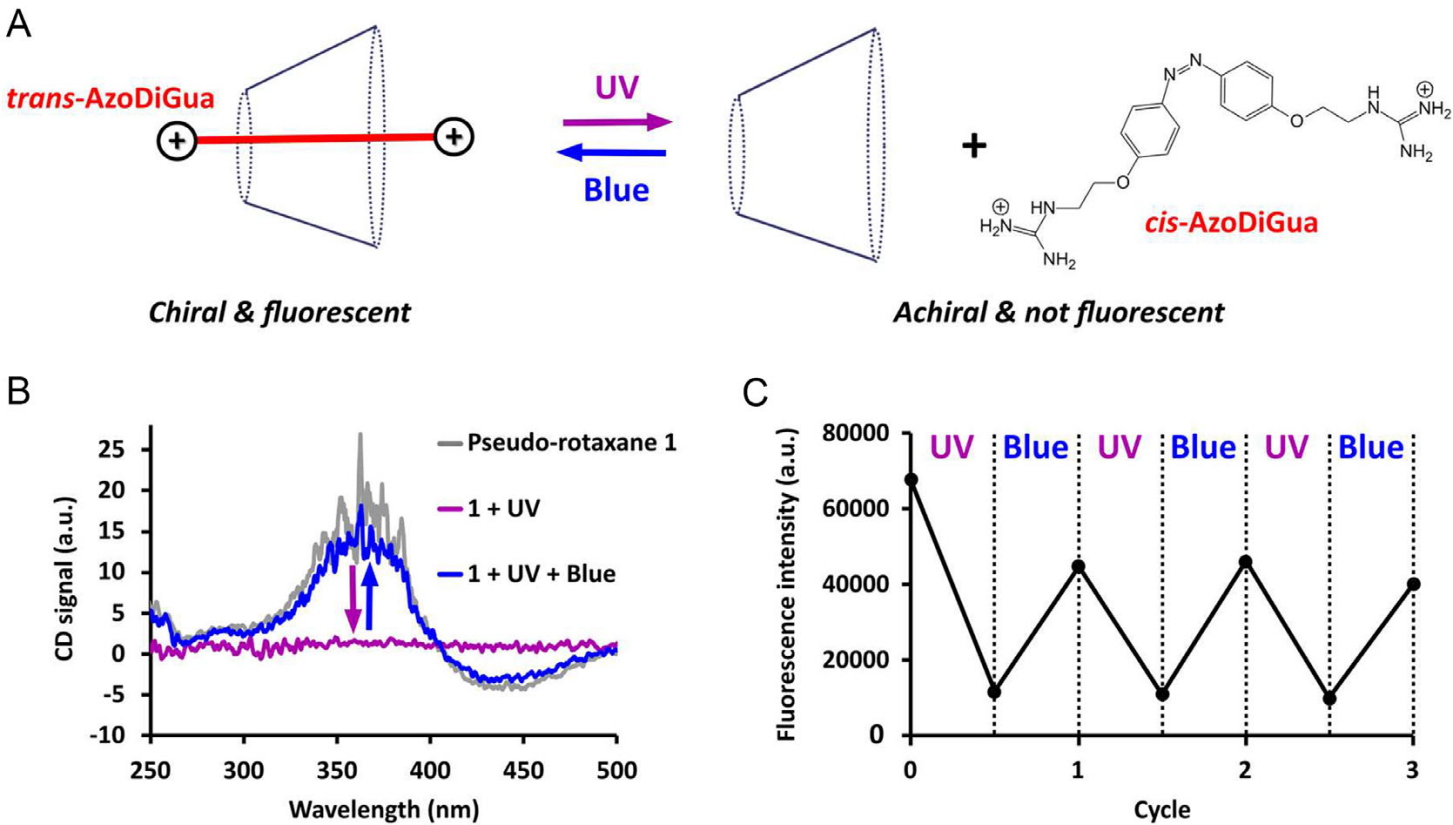
Photoswitchable chirality and fluorescence of pseudo‐rotaxane **1**. A) We hypothesize that the reversible *trans*–*cis* photoisomerization of AzoDiGua results in the photoreversible pseudo‐rotaxane **1** formation/dissociation accompanied by an on/off photoswitchable chirality and fluorescence upon blue/UV irradiation. B) Circular dichroism spectra of **1** before (grey) and after successive irradiation by UV (purple) and blue (blue). C) Fluorescence intensity (Excitation: 400 nm; Emission: 650 nm) of **1** along successive cycles of irradiation by UV and blue light. For all experiments: [AzoDiGua] = 500 μM, [α‐CD] = 500 μM, [Tris HCl] = 50 mM, UV irradiation (5 min at 365 nm, 24 mW cm^−2^), Blue irradiation (5 min at 435 nm, 112 mW cm^−2^).

AzoDiGua is a photoresponsive molecule able to undergo a reversible and fast photoisomerization between *trans* and *cis* conformations under UV and blue irradiations, respectively (Figure S9, Supporting Information).^[^ ^23^, ^32^, ^33^ ^]^ We thus explored the possibility to photocontrol the inclusion‐induced chirality and fluorescence of pseudo‐rotaxane **1** by its photoreversible dissociation and reformation under UV and blue light, respectively (**Figure** **3**A).

When the pseudo‐rotaxane **1** was irradiated with UV light (365 m, 24 mW cm^−2^) for 5 min, AzoDiGua photo‐isomerized into a majority of *cis*‐isomer in the same way as in the absence of α‐CD (Figure S10A, Supporting Information), showing that the initial inclusion of *trans*‐AzoDiGua did not affect its photo‐isomerization characteristics. Interestingly, this was accompanied by a complete vanishing of the circular dichroism signal which became silent after UV irradiation (Figure 3B and S10B, Supporting Information), indicating the disassembly of **1** upon AzoDiGua photo‐isomerization *into* cis isomer and loss of the chirality transfer. When the resulting solution was irradiated with blue light (435 nm, 112 mW cm^−2^) for 5 min, the positive and negative Cotton effects observed before irradiation were recovered (Figure 3B), demonstrating re‐inclusion of *trans*‐AzoDiGua into α‐CD cavity and evidencing efficient photoreversible switching of the inclusion‐induced chirality.

We also investigated the possibility of controlling the fluorescence of the pseudo‐rotaxane **1**. After irradiating **1** with UV light for 5 min, AzoDiGua fluorescence emission (excitation 400 m) decreased drastically (Figure S11, Supporting Information) due to the UV‐induced disassembly of **1** and loss of the fluorescence exaltation observed upon AzoDiGua/α‐CD complexation. Note that the fluorescence emission after irradiating **1** was slightly higher than after irradiating *trans*‐AzoDiGua alone, which can be due to a small fraction of *trans*‐AzoDiGua remaining complexed within α‐CD or to a partial interaction between α‐CD and *cis*‐AzoDiGua. We then exploited this light‐controlled complexation‐induced fluorescence to build a supramolecular fluorescent ON/OFF photoswitch. When **1** was irradiated by a series of consecutive cycles of UV/blue light, its fluorescence emission at 650 nm (excitation 400 nm) reproducibly vanished (UV) and recovered (blue) in a robust and reversible manner (Figure 3C, Supporting Information). All these results show that the photodependent inclusion of AzoDiGua in α‐CD confers to the pseudo‐rotaxane **1** marked light‐responsive properties such as photoswitchable chirality and fluorescence.

### 2.5 Supramolecular Tuning of DNA Melting Temperature

**Figure 4:**
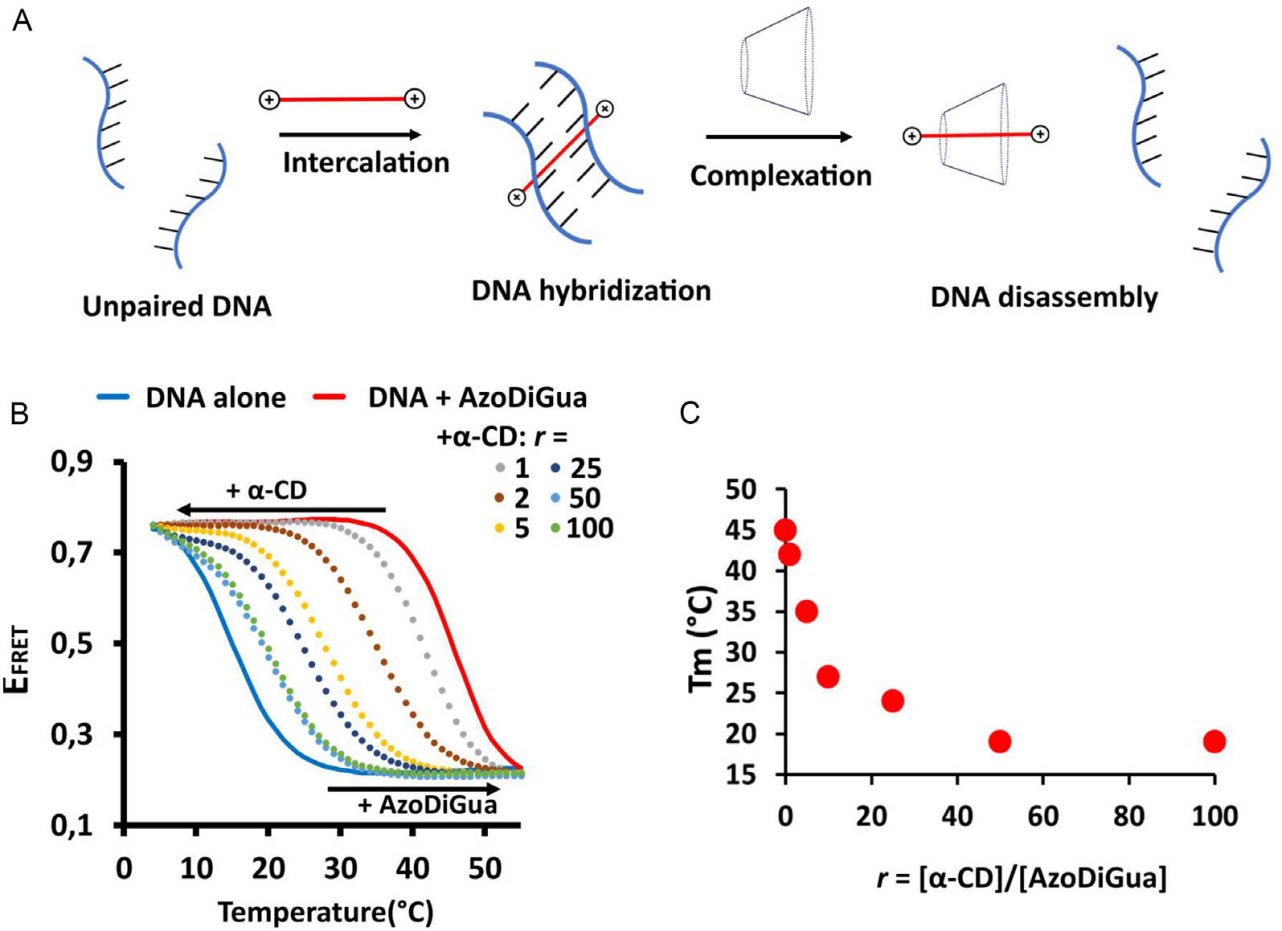
Pseudo‐rotaxane formation as a supramolecular tuning of DNA melting temperature. A) Scheme of α‐CD‐triggered DNA disassembly by the competitive capture of intercalating AzoDiGua and formation of pseudo‐rotaxane **1**. B) FRET efficiency (*E*_FRET_) of a DNA hairpin with a 6‐bp stem (Figure S12, Supporting Information) as a function of temperature without (blue solid line) or with AzoDiGua before (red solid line) and after (colored dotted lines) adding increasing ratios *r* of α‐CD (*r *= [α‐CD]/[AzoDiGua]). C) Melting temperature (*T*_m_) of the DNA hairpin stem stabilized by AzoDiGua, as a function of *r*. All experiments were made in Tris–HCl (10 mM) supplemented with NaCl (75 mM); [DNA hairpin] = 1 μM; [AzoDiGua] = 50 μM.

AzoDiGua is a DNA intercalator that interacts with double‐stranded DNA through π–π stacking and electrostatic interactions between its guanidinium moeities and the phosphate groups of DNA, resulting in a strong stabilization of the double helix against melting.^[^ ^23^ ^]^ We thus investigated the possibility of using α‐CD as a supramolecular stimulus to capture AzoDiGua and tune the melting temperature of DNA (*T*_m_), which could be used, for instance, to trigger DNA disassembly at constant temperature (**Figure** **4**A). As a proof of concept, we used a DNA hairpin system having additional double‐stranded arms enabling close positionning of two fluorophores engaged in a Förster resonance energy transfer (FRET) when the hairpin is in its close state (Figure S12, Supporting Information). This system allowed us to follow the melting of the hairpin stem, a short double strand composed of 6 GC base pairs, through a decrease of the FRET efficiency (*E*_FRET_) (Figure 4B). In our conditions ([Tris–HCl] = 10 mM, [NaCl] = 75 mM), we found that the melting temperature of the stem ([DNA] = 1 μM) drastically increased from *T*_m_ = 16 °C to *T*_m_ = 45 °C upon adding 50 μM of AzoDiGua, confirming the strong stabilization of the DNA double‐helix upon AzoDiGua intercalation. Notably, when increasing amounts of α‐CD was added to the hairpin solution stabilized with AzoDiGua, the *T*_m_ of DNA progressively decreased and reached a value of *T*_m_ = 19 °C at a a ratio *r *= [α‐CD]/[AzoDiGua] equals to 100. In contrast, adding the same amounts of α‐CD to the hairpin alone did not affect its *T*_m_ (Figure S13, Supporting Information). These results show that, with a sufficient excess of α‐CD, AzoDiGua inclusion into α‐CD efficiently competes with its intercalation into DNA, resulting in a capture of AzoDiGua and destabilization of double‐stranded DNA against melting. Strongly concentration‐dependent, this behaviour constitutes, to our knowledge, the first supramolecular tuning of DNA melting temperature using cyclodextin as the trigger and through the formation of a pseudo‐rotaxane.

## 3 Conclusion

In this work, we have described a new pseudo‐rotaxane design combining a photosensitive azobenzene‐derived DNA intercalator, AzoDiGua, and α‐cyclodextrin (α‐CD). This pseudo‐rotaxane was obtained by the inclusion of AzoDiGua in the α‐CD cavity, resulting in azobenzene fluorescence enhancement, chirality transfer and capability to remove intercalated AzoDiGua from DNA double helix. Azobenzene photoisomerization was maintained in this system, allowing us to photoreversibly dissociate/associate the pseudo‐rotaxane upon UV/blue irradiation, resulting in a dynamic supramolecular complex with photoswitchable fluorescence and chirality. This work not only highlights some fundamental aspects of reversible inclusion/complexation mechanisms, but can also serve as a guideline to develop a variety of functional dynamic supramolecular platforms. The AzoDiGua inclusion in α‐CD inclusion efficiently competing with its intercalation into DNA, the melting temperature of DNA could be adjusted by gradual addition of α‐CD and pseudo‐rotaxane formation. This creates ground for supramolecular control of DNA hybridization/melting at a fixed temperature, with potential applications from dynamic DNA nanotechnology^[^ ^18^, ^35^, ^36^ ^]^ to biosensing^[^ ^37^, ^38^ ^]^ and therapy.^[^ ^39^, ^40^ ^]^

## Conflict of Interest

The authors declare no conflict of interest.

## Supporting information

Supplementary Material

## Data Availability

The data that support the findings of this study are available from the corresponding author upon reasonable request.
