# Supplementary material for: Azobenzene DNA Intercalator/Cyclodextrin Pseudo‐Rotaxane: From Photoswitchable Chirality and Fluorescence to DNA Melting Control
Source: ChemistryOpen. 2025 Aug 20;14(12):e202500382. doi: 10.1002/open.202500382 (PMC12680564; doi:10.1002/open.202500382)
Supplement: Supplementary file 1 — Supplementary Material [file OPEN-14-e202500382-s001.pdf]

Supporting Information for:

Azobenzene DNA Intercalator/Cyclodextrin Pseudo-Rotaxane: from Photoswitchable Chirality and Fluorescence to DNA Melting Control

Olivier Abodja[1], Astrid Walrant[1], Sergii Rudiuk[1], Mathieu Morel[1], Damien Baigl[1]*

[1]CPCV, Department of Chemistry, Ecole Normale Supérieure, PSL University, Sorbonne Université, CNRS, Paris 75005, France

* Correspondence to : damien.baigl@ens.psl.eu

Contents :

1. Materials and methods
2. Supplementary Figures S1 – S13
3. Supplementary References

1. Materials and methods

Materials

α-Cyclodextrin, sodium chloride and Trizma chloride buffer solution were purchased from Sigma Aldrich. AzoDiGua was used as synthesized according to our previous synthesis procedure[2].

UV-vis spectroscopy

Absorption measurements were performed with and Eppendorf BioSpectrometer with a 10 mm-wide cuvette. All solutions were buffered with Tris HCl (50 mM, pH=7.4).

Fluorescence spectroscopy

Fluorescence measurements were performed on a multimode microplate reader (Spark, Tecan). The solutions were prepared in 96-well microplates and buffered with Tris HCl (50 mM, pH=7.4). Emission spectra were acquired between 500 nm and 800 nm, with a 400 nm excitation wavelength.

Circular dichroism spectroscopy

Circular dichroism spectra were acquired with a JASCO J815 spectrometer at room temperature (T=20°C) with a scanning rate of 200 nm/min in a 1 mm-wide quartz cuvette.

NMR spectroscopy

The 1D and 2D ^{1}H NMR spectra were performed with Bruker NMR spectrometer (300 MHz). D_2O was used as a solvent. The solutions were prepared in NMR tubes and sonicated for 3 minutes for homogenization before acquisition on Topspin software.

Isothermal titration calorimetry

The thermograms were acquired with a NanoITC calorimeter (TA instruments). The measurement was done at T = 25°C. The measurement cell was first washed with water and Tris HCl buffer and then filled with an AzoDiGua solution (1 mM) with a total volume of 1 mL. The injection syringe was filled an α-CD or β-CD solution (10 mM) with a total volume of 250 µL. The two solutions were buffered with Tris HCl (50 mM). After thermal equilibration of the calorimeter, 10 µL injections (2 µL for the first injection) of the α-CD or β-CD solution into the AzoDiGua solution were performed with a time interval of 5 minutes between each injection with a total of 25 injections under mechanical agitation (250 rpm).

Photocontrol study

All irradiations were performed with a fiber-coupled LED source (pE-2, CoolLED, UV irradiation : 365 nm, 24 mW.cm $^{-2}$, Blue irradiation : 435 nm, 112 mW.cm $^{-2}$). The irradiation was performed for 5 minutes and the spectra (fluorescence and circular dichroism) were acquired immediately after. UV irradiation was performed within a dark box protected from visible light.

DNA FRET melting curves

The FRET melting curves were acquired on a qPCR QuantStudio5 machine, following a method similar to a previously published protocol[2]. The solutions were prepared on ice for a total volume of 20 µL in a qPCR multi-well plate ([Tris HCl] = 10 mM, [AzoDiGua] = 50 µM , [NaCl] = 75 mM, [DNA platform] = 1 µM and varying concentrations of α-CD). After mixing the solution, the fluorescence emission intensity of the acceptor and donor groups were respectively measured at 550 nm and 682 nm for a 520 nm excitation wavelength. The fluorescence intensities were measured every 1 °C from 4°C to 97°C (heating rate of 1°C/5 s). The hairpin sequence contains 6 GC base pairs (Figure S12). The FRET efficiency was measured as :

$$E_{FRET} = \frac{I_A}{I_A + I_B}$$

With I_A and I_B the fluorescence intensity of the acceptor and receptor, respectively.

2. Supplementary Figures

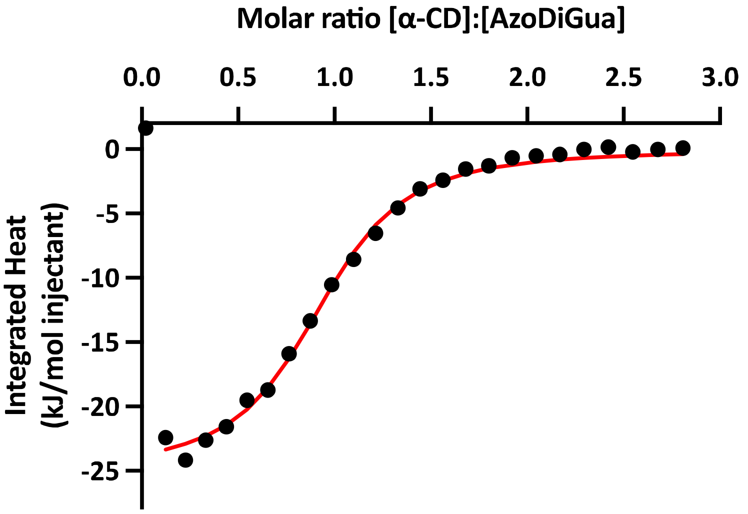

Figure S1. Isothermal titration calorimetry (ITC) thermogram exploited-curve for AzoDiGua/ α-CD complexation ([AzoDiGua] = 1 mM in measurement cell, [α-CD] = 10 mM in syringe, T = 25°C). Experiments were performed in Tris HCl buffer (50 mM).

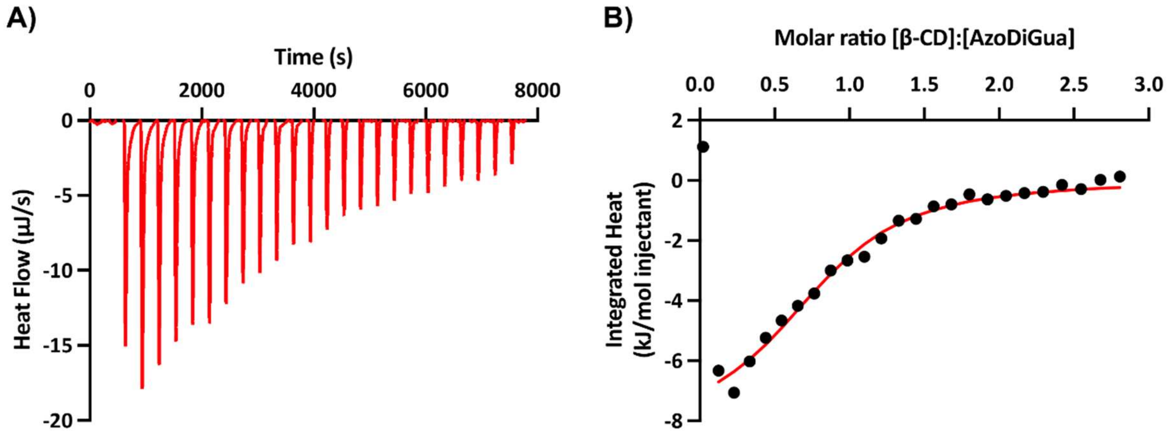

Figure S2. Isothermal titration calorimetry (ITC) thermogram (A) and thermogram exploited-curve (B) for AzoDiGua/ β-CD complexation ([AzoDiGua] = 1 mM in measurement cell, [β-CD] = 10 mM in syringe, T = 25°C). Experiments were performed in Tris HCl buffer (50 mM).

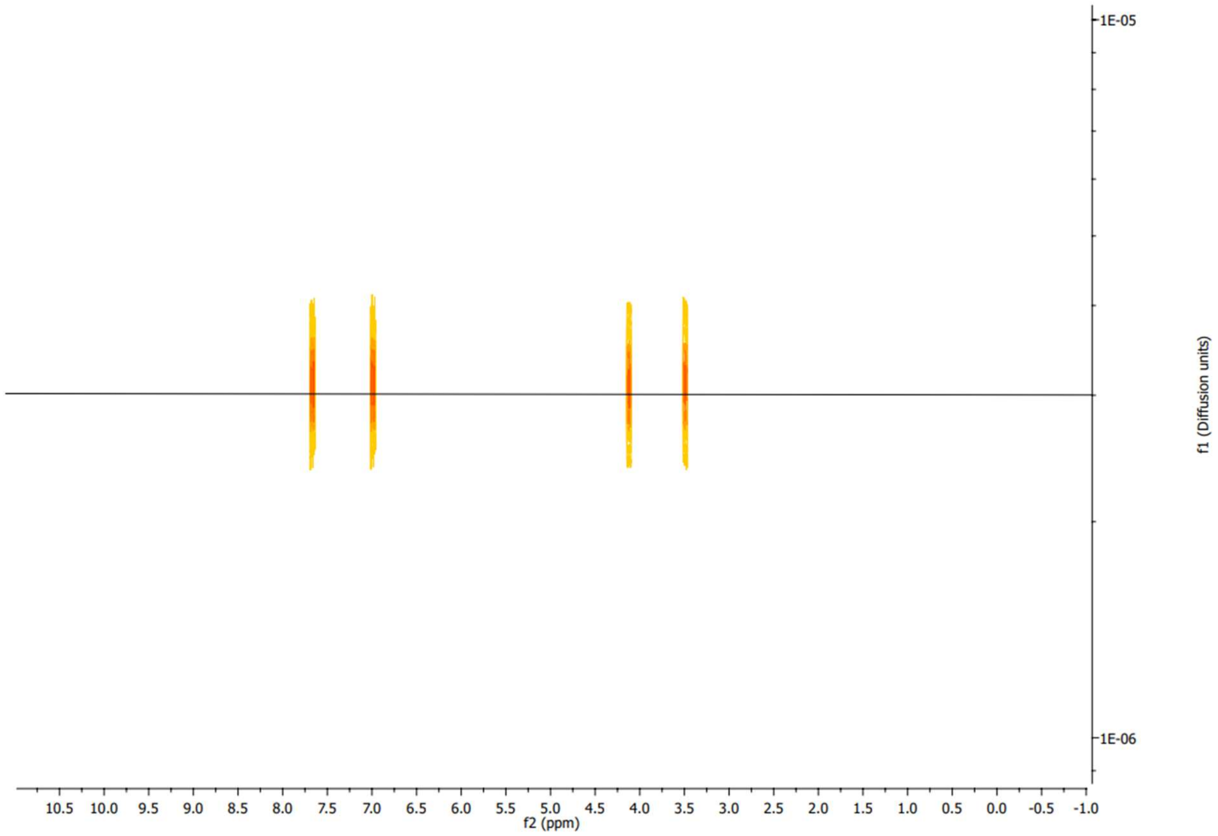

Figure S3. 2D DOESY ^{1}H NMR spectrum of AzoDiGua (300 MHz, D_2O, [AzoDiGua] = 5 mM). The horizontal line indicates a diffusion coefficient of $3\cdot10^{-6}$ $m^2\cdot s^{-1}$.

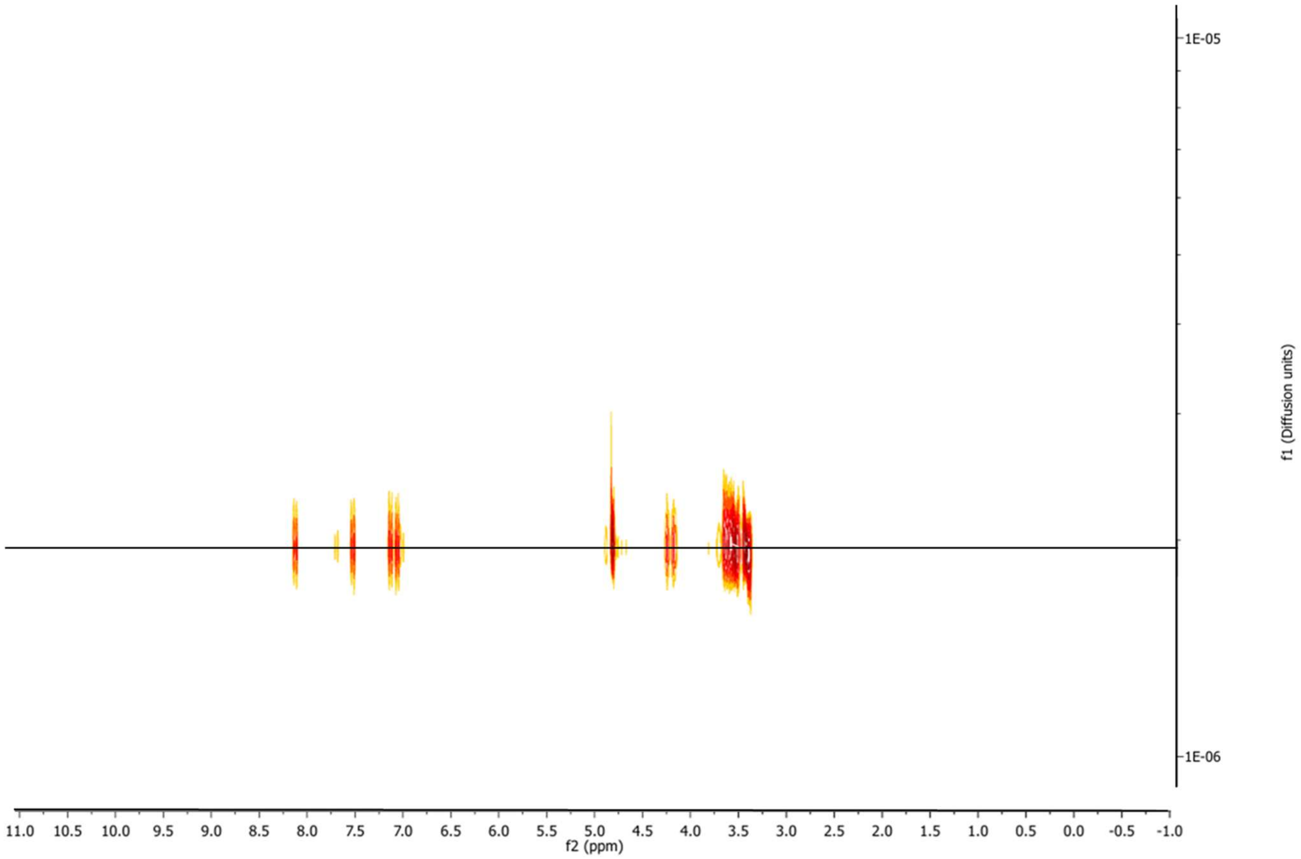

Figure S4. 2D DOSY ^{1}H NMR spectrum of an equimolar mixture of AzoDiGua and α-CD (300 MHz, D_2O, [AzoDiGua] = [α-CD] = 5 mM). The horizontal line indicates a diffusion coefficient of $1.9 \cdot 10^{-6}$ $m^2 \cdot s^{-1}$.

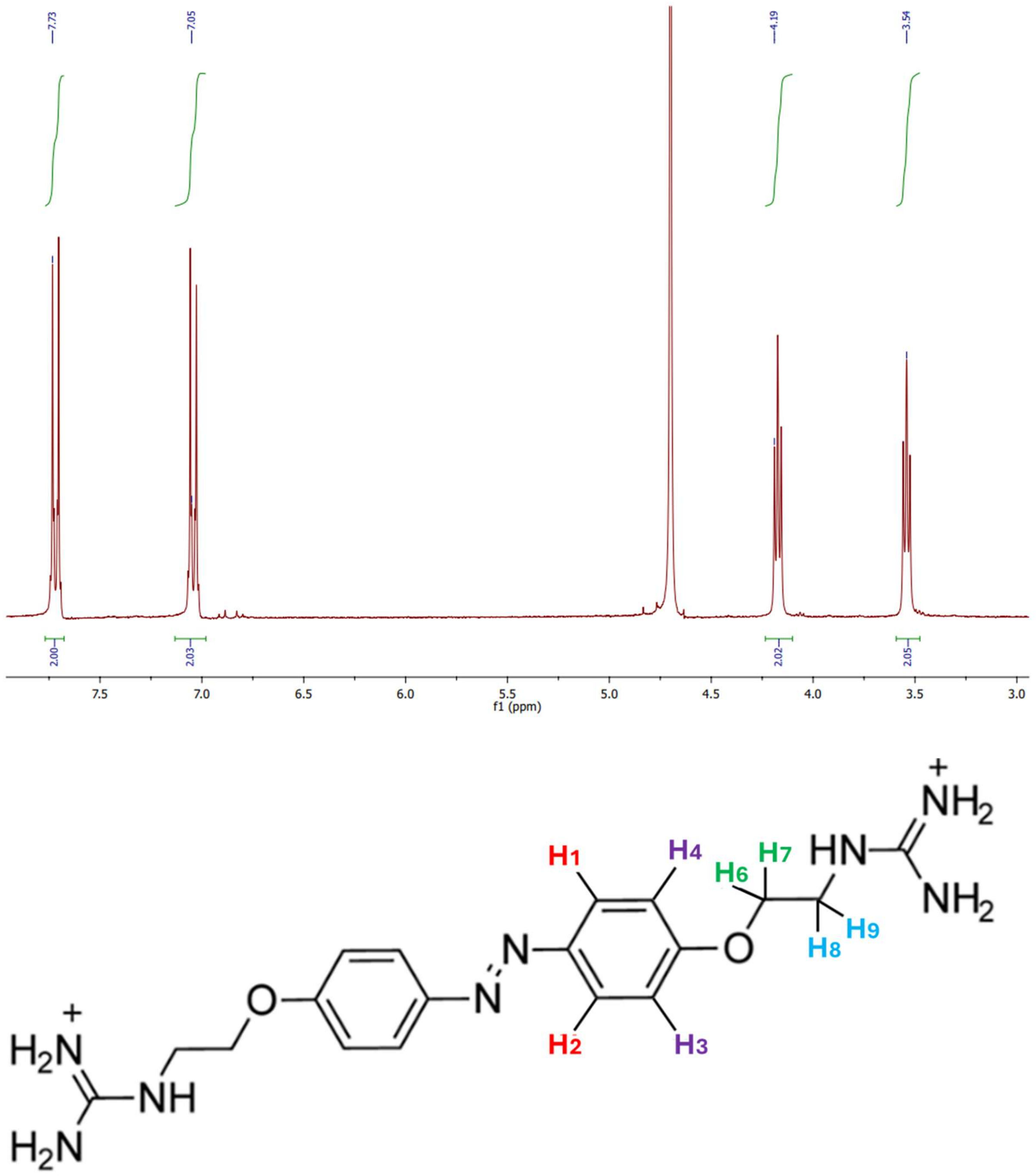

Figure S5. *Top.* ^{1}H NMR spectrum of AzoDiGua (300 MHz, D_2O, [AzoDiGua] = 5 mM). *Bottom.* AzoDiGua molecular structure with proton assignments. ^{1}H NMR (300 MHz, D_2O): 4.81 (H_2O), 7.73 (doublet, 2H, H_3-H_4), 7.05 (doublet, 2H, H_1-H_2), 4.19 (triplet, 2H, H_6-H_7), 3.54 (triplet, 2H, H_8-H_9).

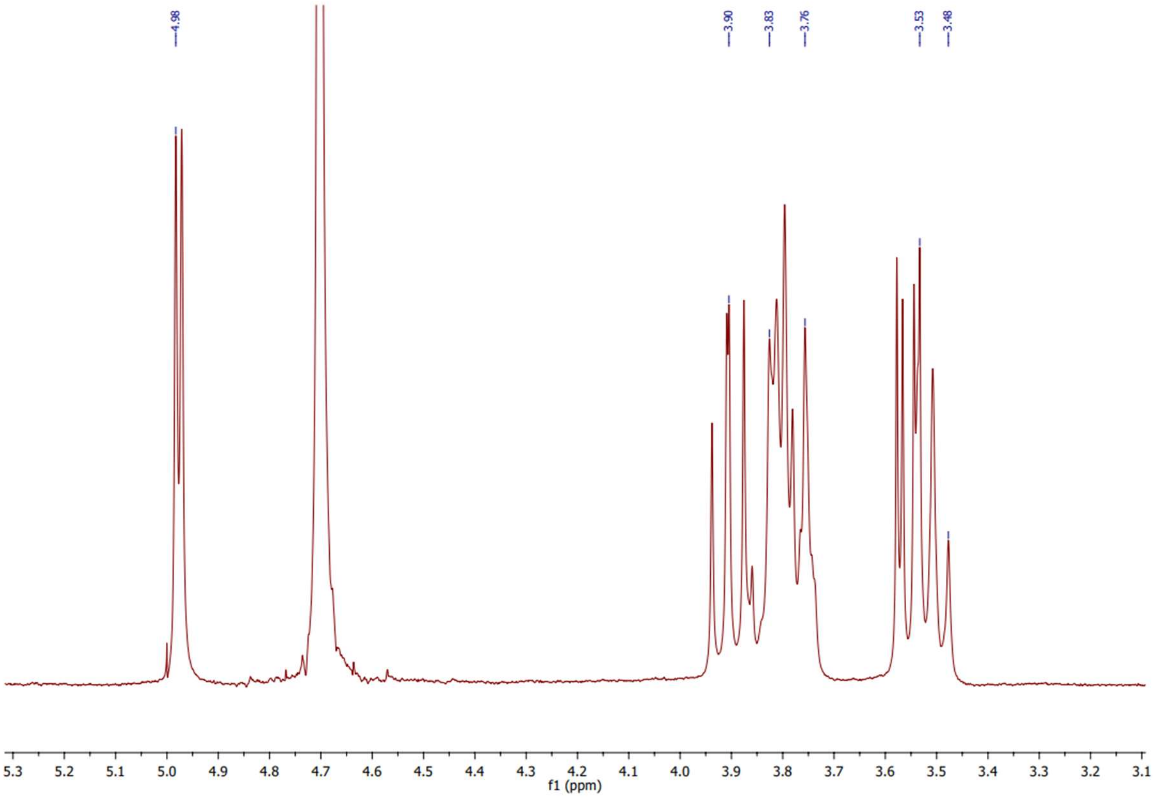

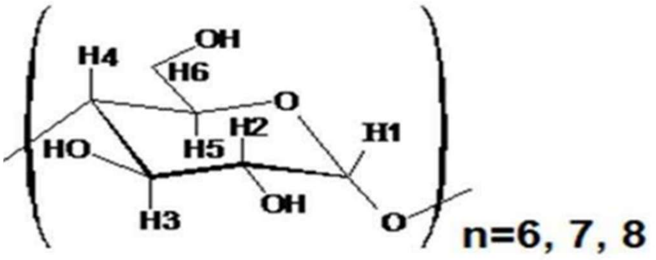

Figure S6. *Top*. ^{1}H NMR spectrum of α-CD (300 MHz, D_2O, [α-CD] = 5 mM). *Bottom.* α-CD molecular structure with proton assignments according to literature.[1] ^{1}H NMR (300 MHz, D_2O): 4.98 (H1), 3.90 (H3), 3.83 (H5), 3.76 (H6), 3.53 (H2), 3.48 (H4).

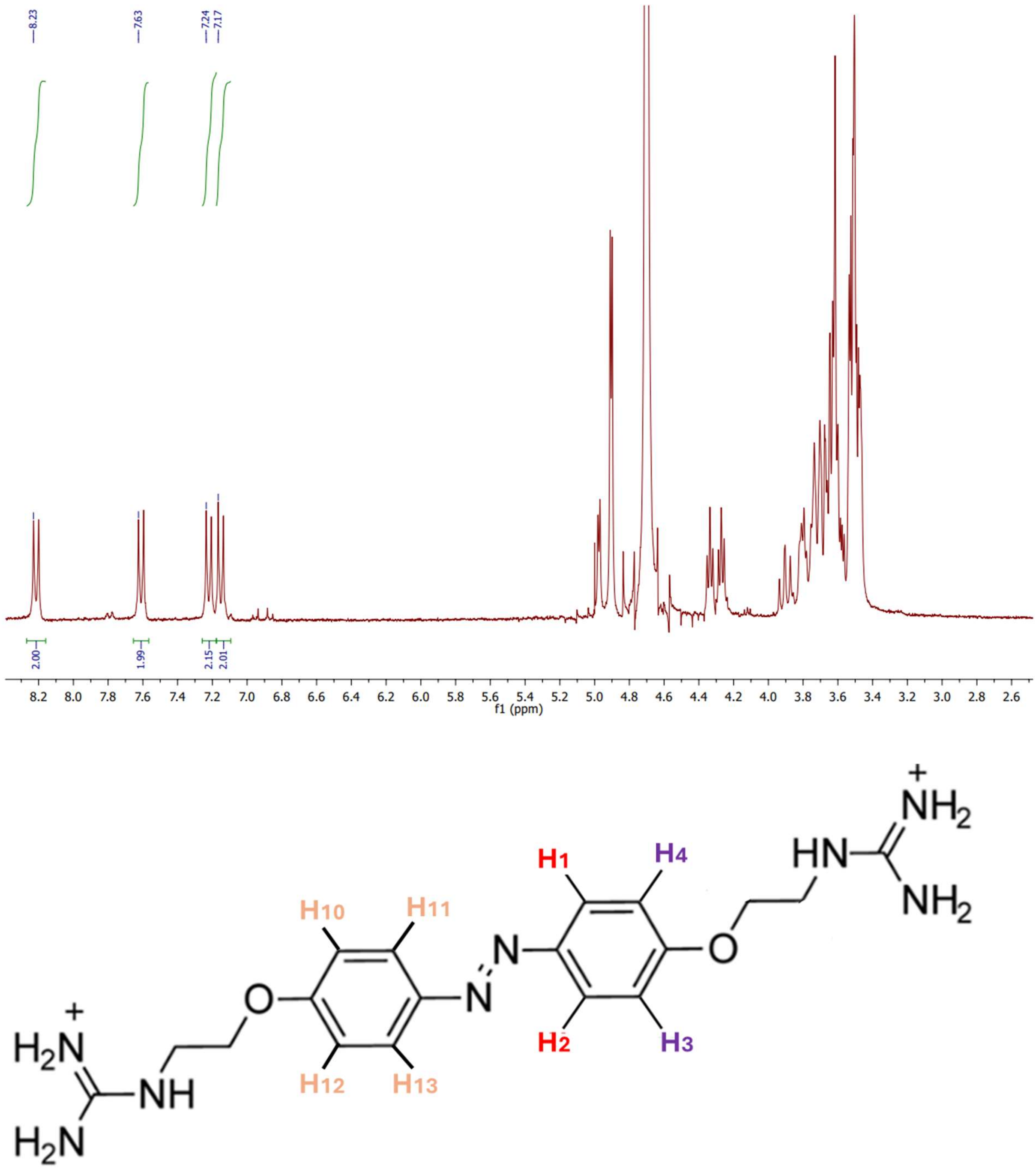

Figure S7. *Top.* ^{1}H NMR spectrum of an equimolar mixture of AzoDiGua and α-CD (300 MHz, D_2O, [AzoDiGua] = [α-CD] = 5 mM). *Bottom.* AzoDiGua molecular structure with proton assignments. ^{1}H RMN (300 MHz, D_2O): 8.23 (doublet, 2H, H_3-H_4), 7.63 (doublet, 2H, H_1-H_2); 7.24-7.17 (two doublets, 2H,2H, H_{10}-H_{13}).

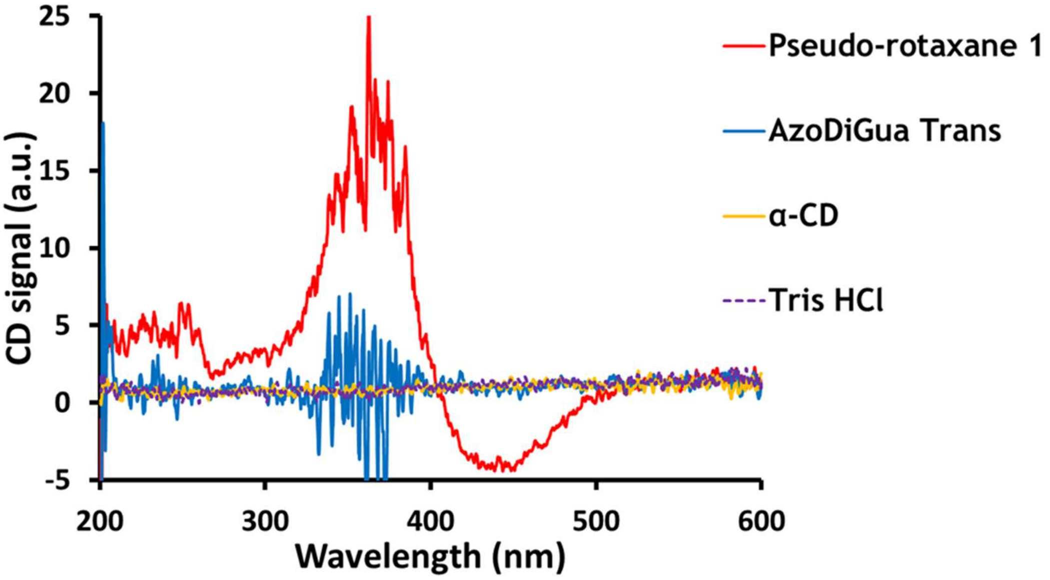

Figure S8. Circular dichroism spectra of Tris HCl, α-CD in Tris-HCl, AzoDiGua in Tris-HCl or the pseudo-rotaxane **1**. [α-CD] = 500 μM; [AzoDiGua] = 500 μM; [Tris HCl] = 50 mM.

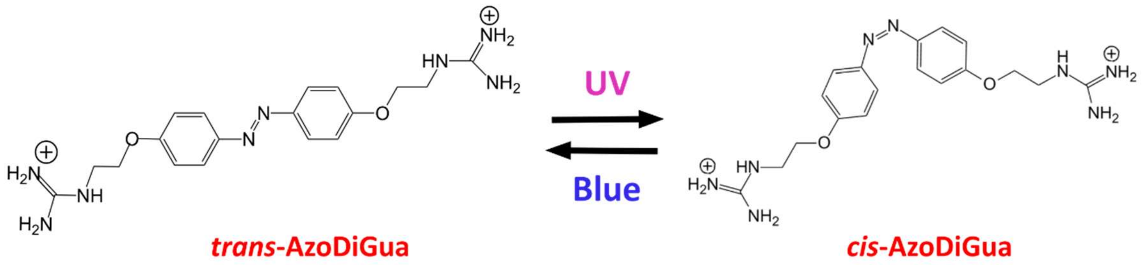

Figure S9. Photoreversible *trans-cis* isomerization of AzoDiGua.

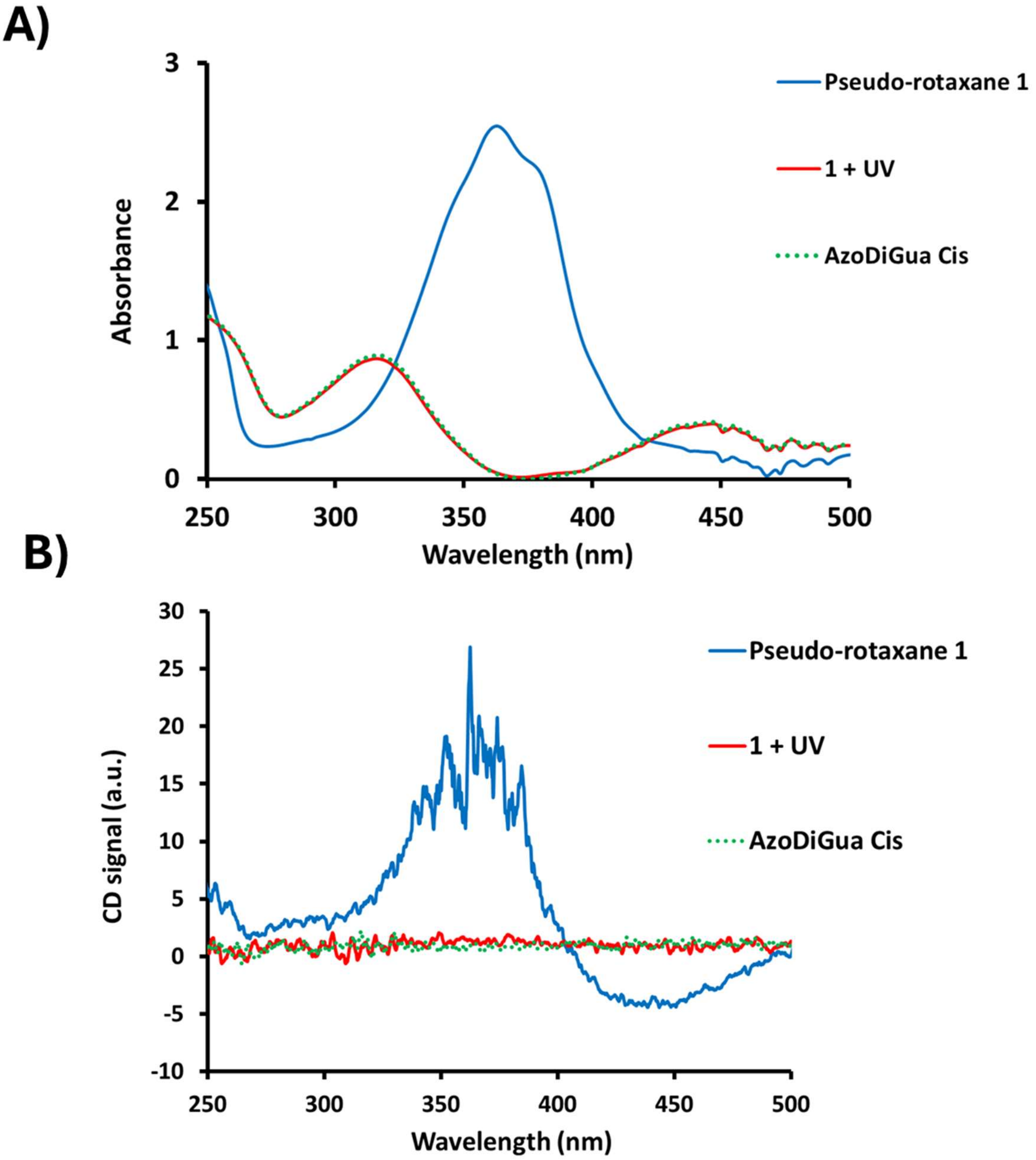

Figure S10. UV-Vis absorbance (A) and circular dichroism (B) spectra of pseudo-rotaxane **1** before and after UV irradiation, as well as *cis*-AzoDiGua alone obtained from *trans*-AzoDiGua subjected to the same UV irradiation. For all experiments: [α-CD] = 500 µM in 50 mM Tris HCl; [AzoDiGua] = 500 µM in Tris-HCl; UV irradiation (365 nm, 24 mW.cm $^{-2}$ for 5 min).

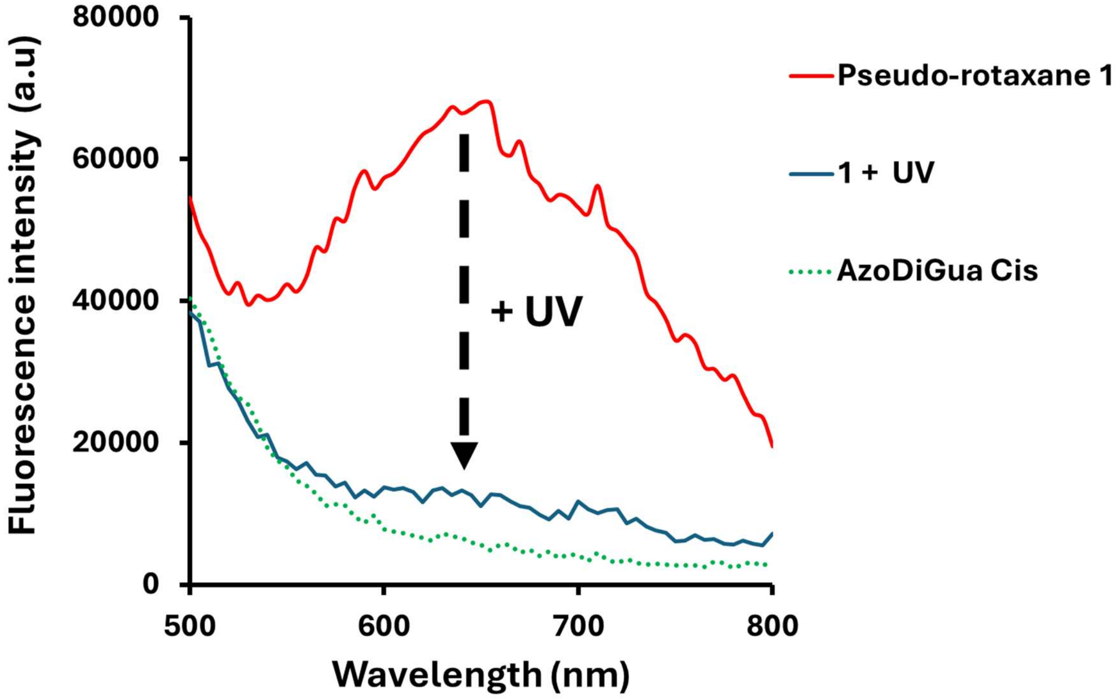

Figure S11. Fluorescence emission spectrum (Excitation: 400 m) of **1** before and after UV irradiation, as well as *cis*-AzoDiGua alone obtained from *trans*-AzoDiGua subjected to the same UV irradiation. For all experiments: [α-CD] = 500 µM in 50 mM Tris HCl; [AzoDiGua] = 500 µM in Tris-HCl; UV irradiation (365 nm, 24 mW.cm $^{-2}$ for 5 min).

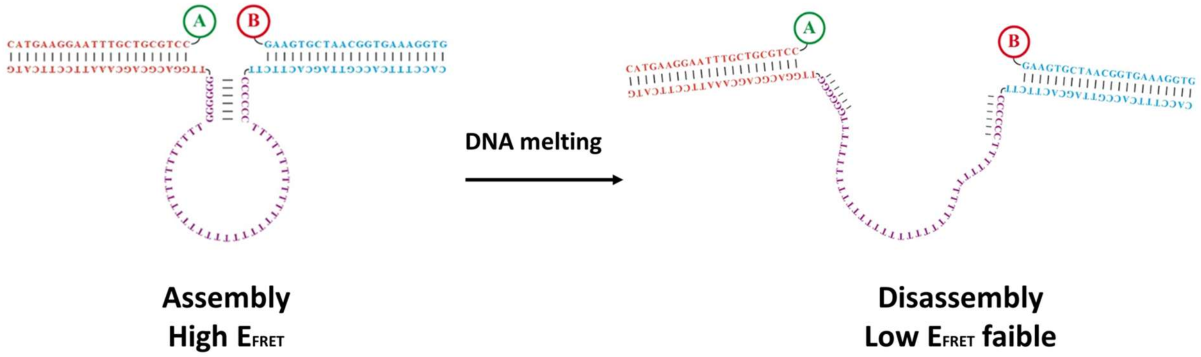

Figure S12. Schematic scheme of a DNA hairpin DNA modified with a Förster resonance energy transfer (FRET) acceptor (A, Cy5) and donor (B, CY3). The FRET efficiency (E_{FRET}) is high (respectively low) when the hairpin is the close (respectively open) state (see Materials and methods for details). Measuring E_{FRET} is thus a way to follow the melting of the 6-bp DNA stem (here 100% GC) separating the fluorophore-modified arms from the loop.

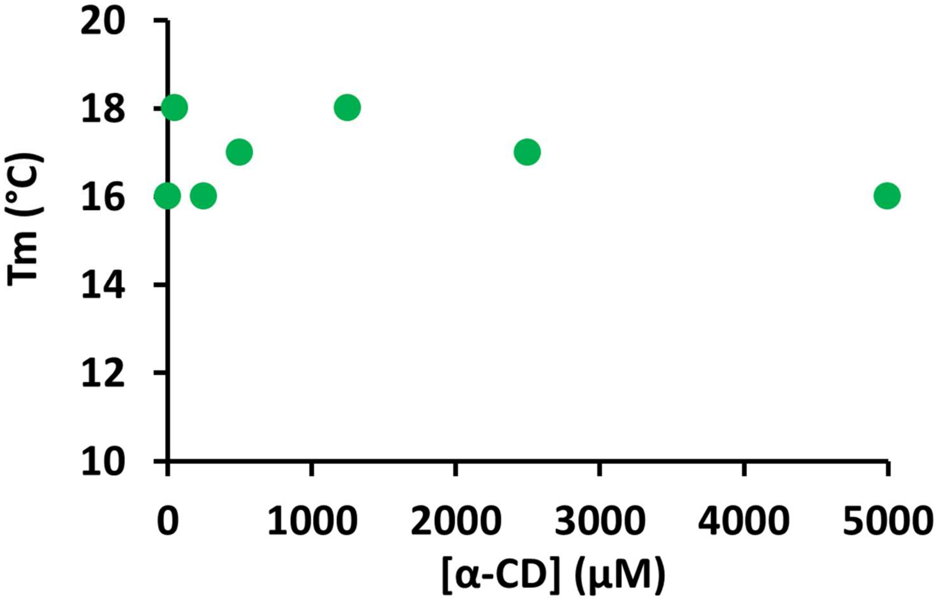

Figure S13. Evolution of the melting temperature (T_m) of the hairpin stem (**Figure S12**), ([DNA] = 1 µM) with increasing concentrations of α-CD. The solution are buffered in Tris HCl ([Tris HCl] = 10 mM) and contains 75 mM NaCl.

3. Supplementary References

(1) T. Pessine, F. B.; Calderini, A.; L., G. Review: Cyclodextrin Inclusion Complexes Probed by NMR Techniques. *Magn. Reson. Spectrosc.* **2012**, *1*. https://doi.org/10.5772/32029.

(2) Bergen, A.; Rudiuk, S.; Morel, M.; Le Saux, T.; Ihmels, H.; Baigl, D. Photodependent Melting of Unmodified DNA Using a Photosensitive Intercalator: A New and Generic Tool for Photoreversible Assembly of DNA Nanostructures at Constant Temperature. *Nano Lett.* **2016**, *16* (1), 773–780. https://doi.org/10.1021/acs.nanolett.5b04762.
